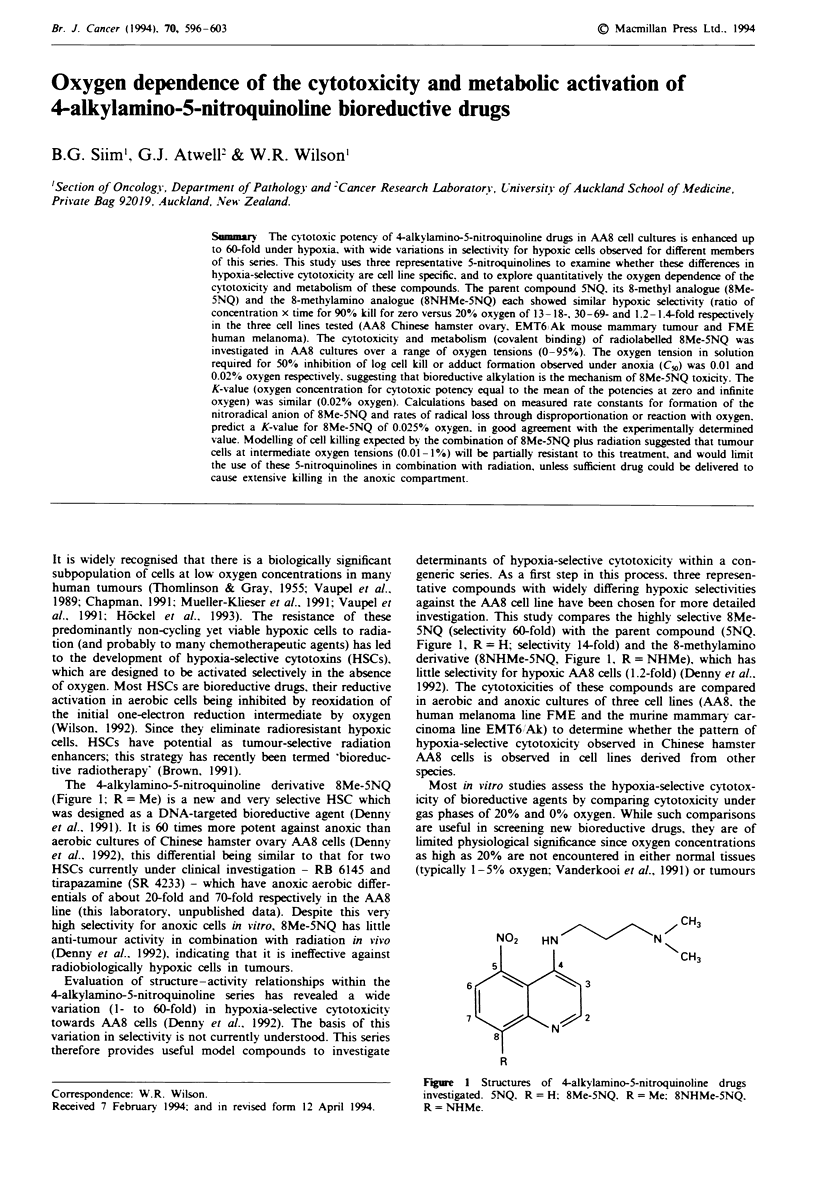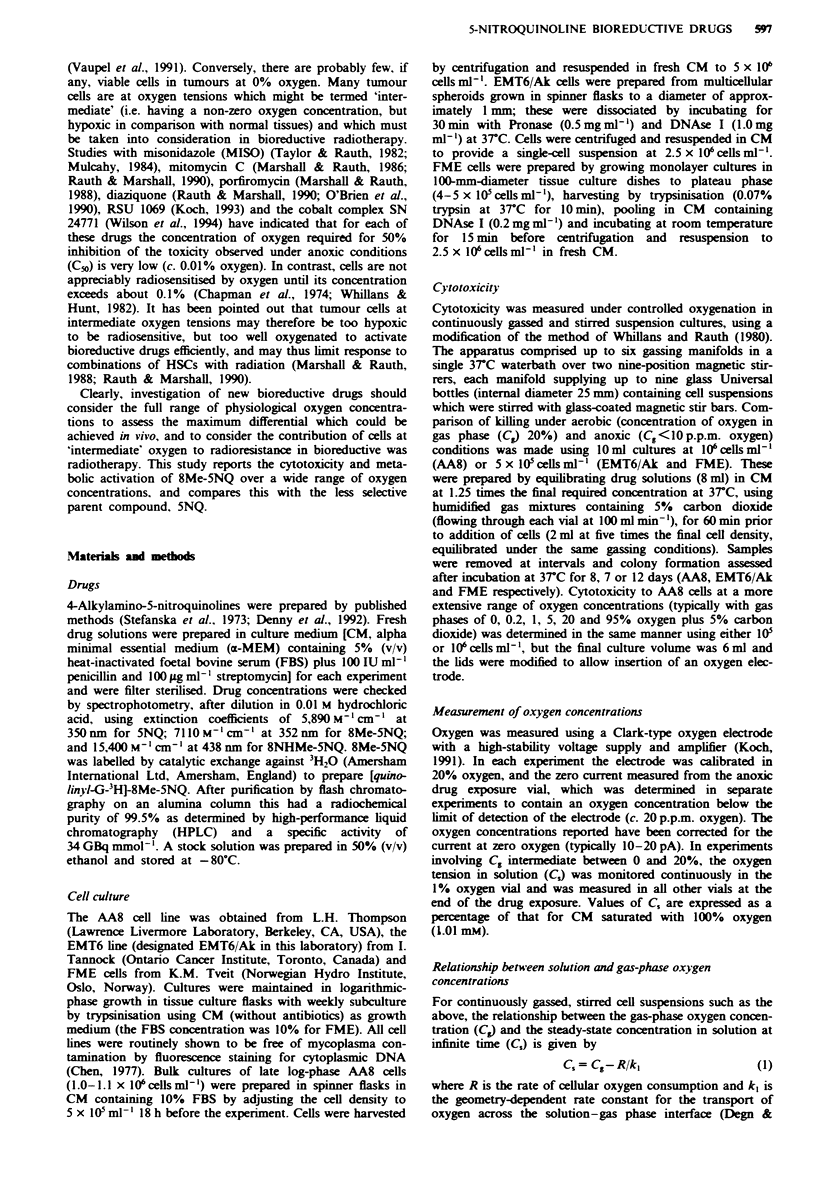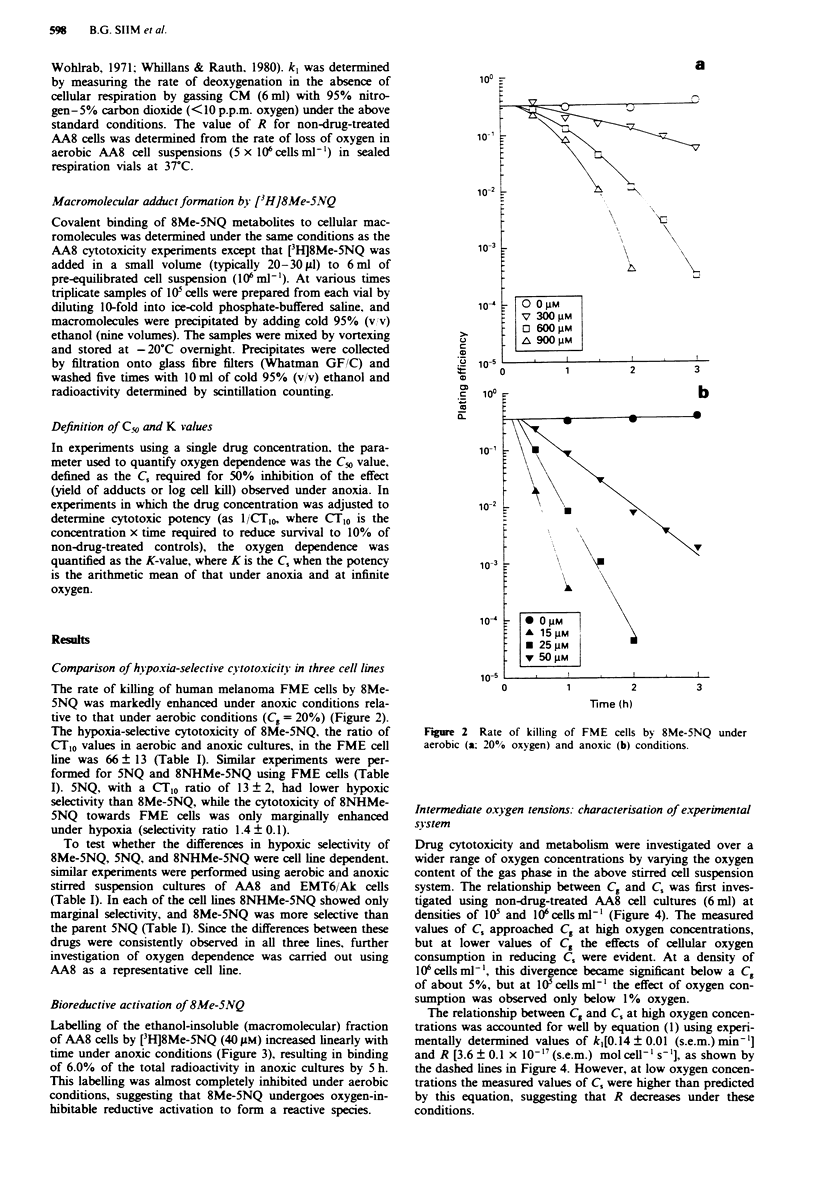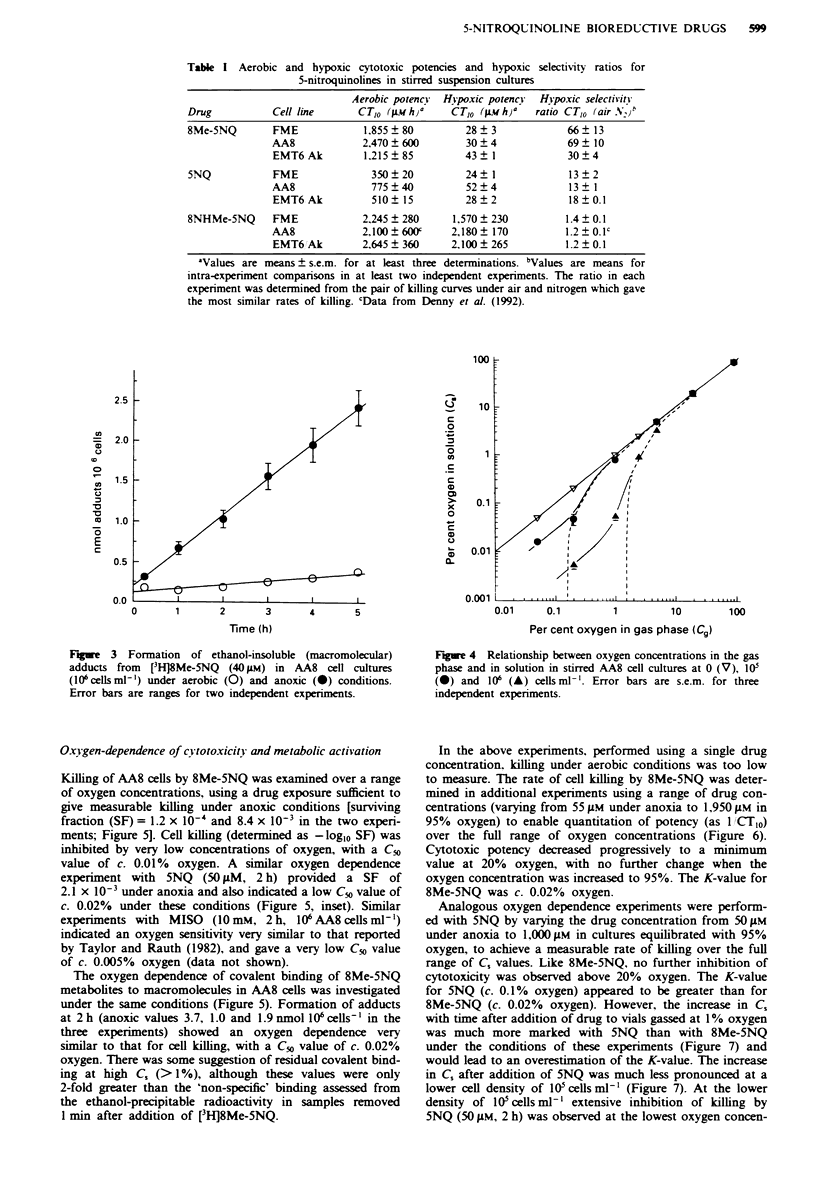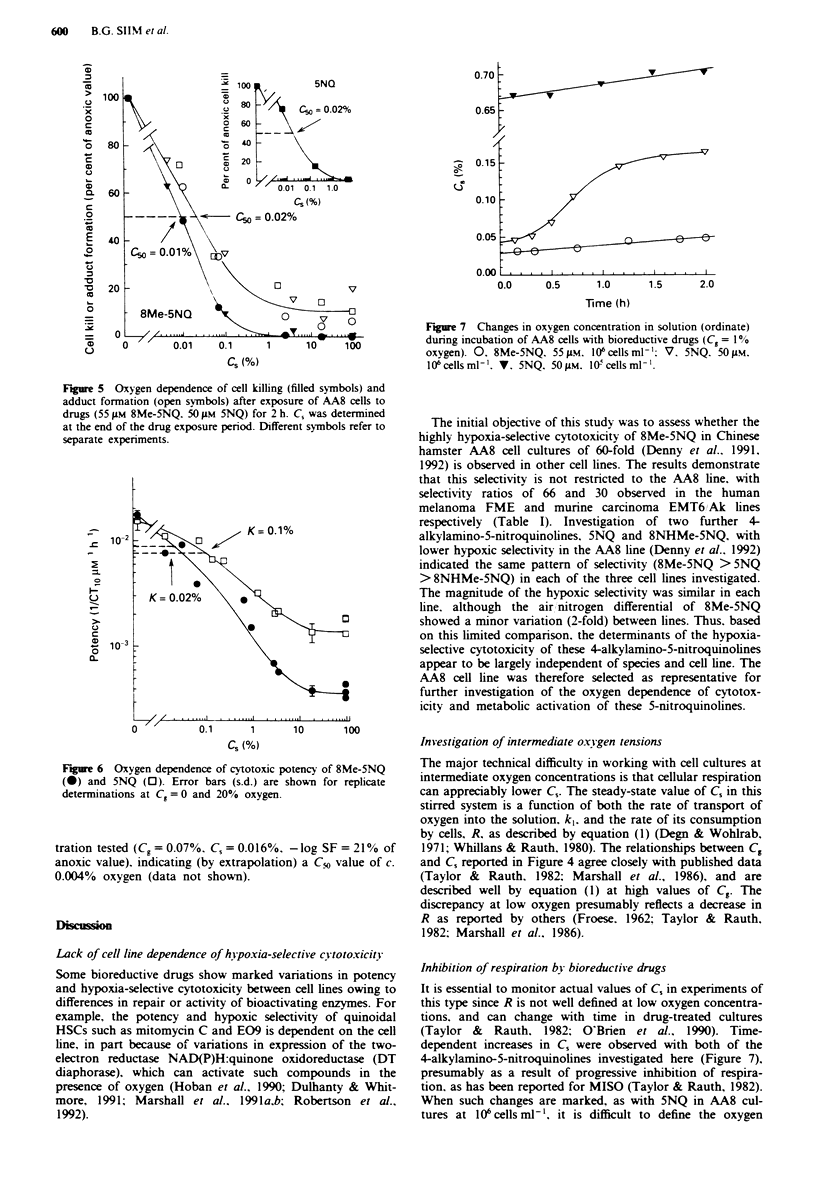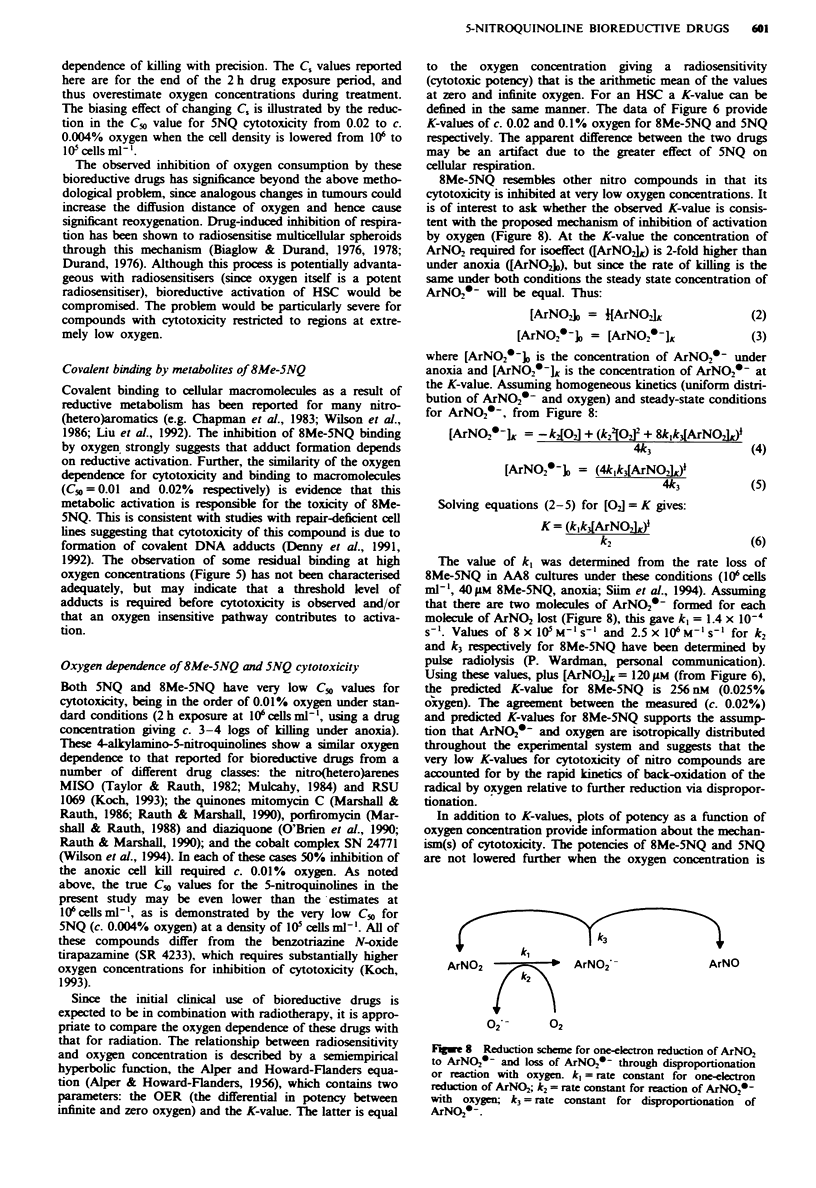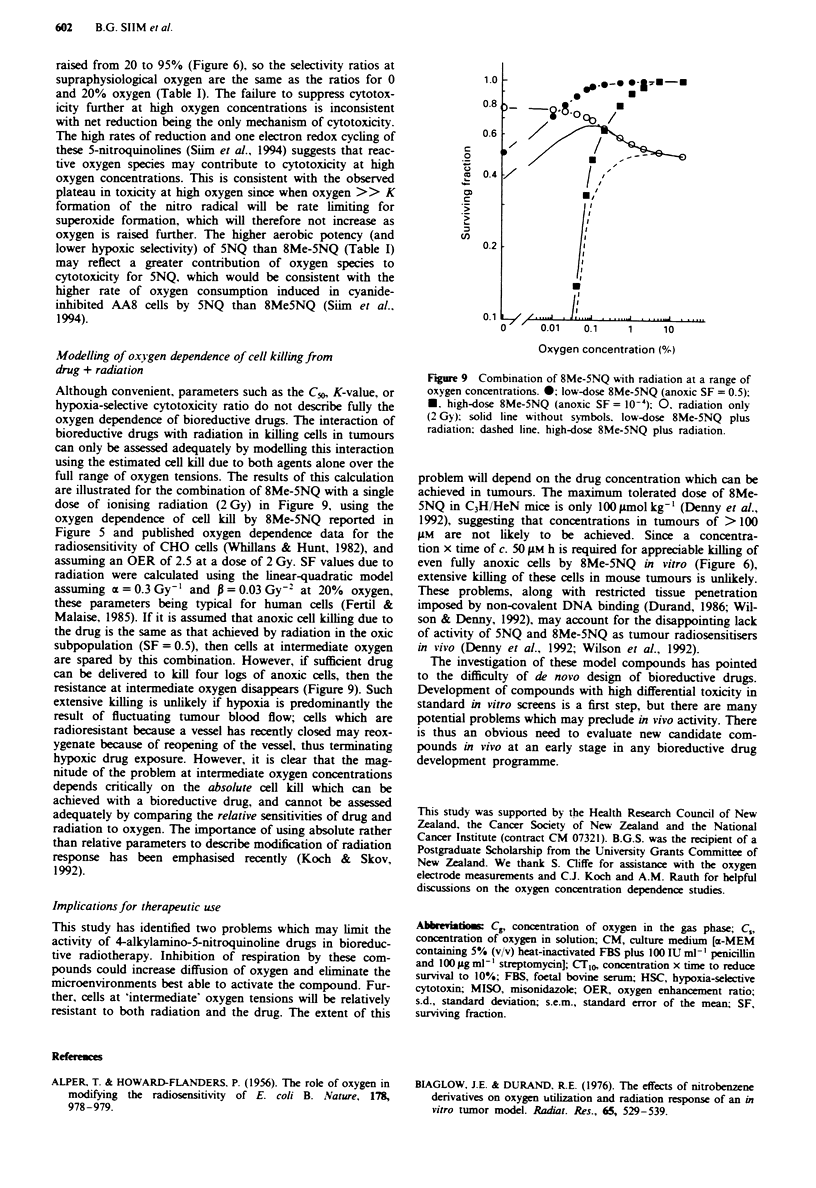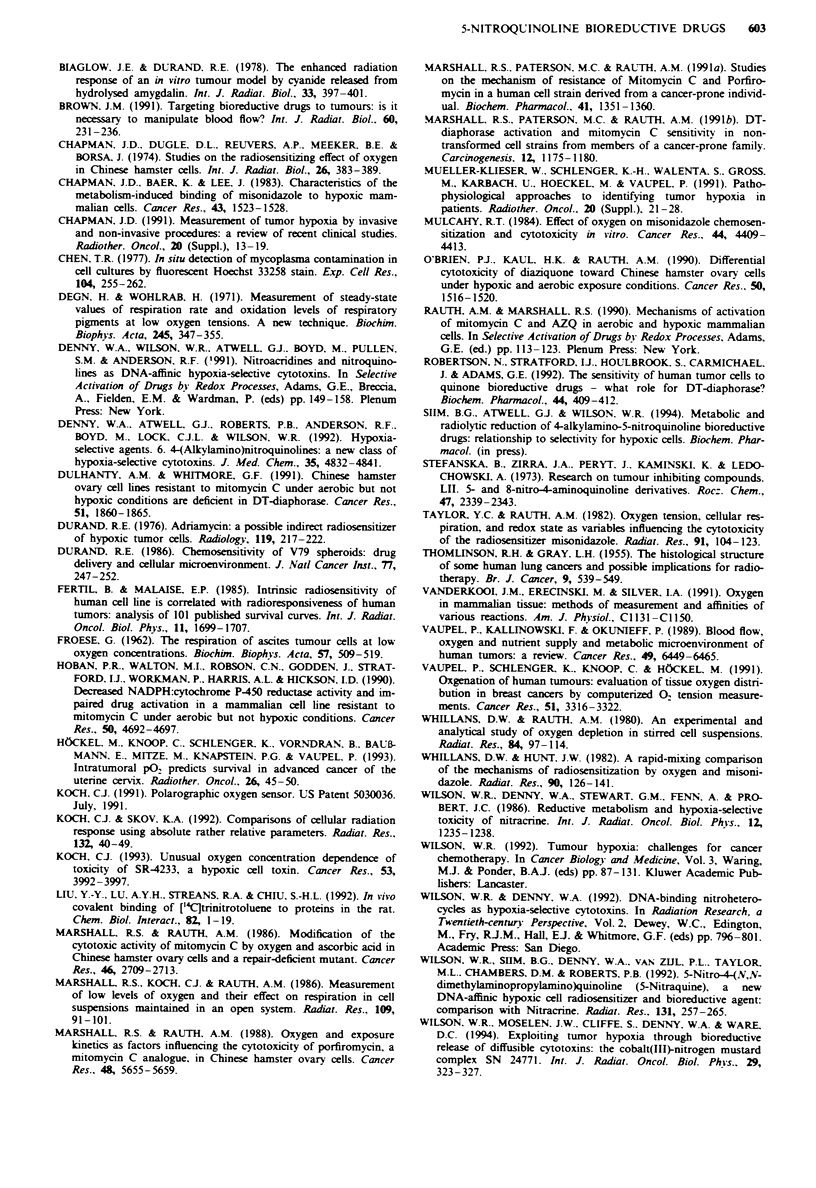# Oxygen dependence of the cytotoxicity and metabolic activation of 4-alkylamino-5-nitroquinoline bioreductive drugs.

**DOI:** 10.1038/bjc.1994.357

**Published:** 1994-10

**Authors:** B. G. Siim, G. J. Atwell, W. R. Wilson

**Affiliations:** Department of Pathology, University of Auckland School of Medicine, New Zealand.

## Abstract

The cytotoxic potency of 4-alkylamino-5-nitroquinoline drugs in AA8 cell cultures is enhanced up to 60-fold under hypoxia, with wide variations in selectivity for hypoxic cells observed for different members of this series. This study uses three representative 5-nitroquinolines to examine whether these differences in hypoxia-selective cytotoxicity are cell line specific, and to explore quantitatively the oxygen dependence of the cytotoxicity and metabolism of these compounds. The parent compound 5NQ, its 5NQ, its 8-methyl analogue (8Me5NQ) and the 8-methylamino analogue (8NHMe-5NQ) each showed similar hypoxic selectivity (ratio of concentration x time for 90% kill for zero versus 20% oxygen of 13-18-, 30-69- and 1.2-1.4-fold respectively in the three cell lines tested (AA8 Chinese hamster ovary, EMT6/Ak mouse mammary tumour and FME human melanoma). The cytotoxicity and metabolism (covalent binding) of radiolabelled 8Me-5NQ was investigated in AA8 cultures over a range of oxygen tensions (0-95%). The oxygen tension in solution required for 50% inhibition of log cell kill or adduct formation observed under anoxia (C50) was 0.01 and 0.02% oxygen respectively, suggesting that bioreductive alkylation is the mechanism of 8Me-5NQ toxicity. The K-value (oxygen concentration for cytotoxic potency equal to the mean of the potencies at zero and infinite oxygen) was similar (0.02% oxygen). Calculations based on measured rate constants for formation of the nitroradical anion of 8Me-5NQ and rates of radical loss through disproportionation or reaction with oxygen, predict a K-value for 8Me-5NQ of 0.025% oxygen, in good agreement with the experimentally determined value. Modelling of cell killing expected by the combination of 8Me-5NQ plus radiation suggested that tumour cells at intermediate oxygen tensions (0.01-1%) will be partially resistant to this treatment, and would limit the use of these 5-nitroquinolines in combination with radiation, unless sufficient drug could be delivered to cause extensive killing in the anoxic compartment.


Br. J. Cancer (1994), 70, 596-603 © Macmillan Press Ltd., 1994

### Oxygen dependence of the cytotoxicity and metabolic activation of 4-alkylamino-5-nitroquinoline bioreductive drugs

B.G. Siim[1], G.J. Atwell[2] & W.R. Wilson[1]

[1]Section of Oncology, Department of Pathology and [2]Cancer Research Laboratory, University of Auckland School of Medicine, Private Bag 92019, Auckland, New Zealand.

**Summary** The cytotoxic potency of 4-alkylamino-5-nitroquinoline drugs in AA8 cell cultures is enhanced up to 60-fold under hypoxia, with wide variations in selectivity for hypoxic cells observed for different members of this series. This study uses three representative 5-nitroquinolines to examine whether these differences in hypoxia-selective cytotoxicity are cell line specific, and to explore quantitatively the oxygen dependence of the cytotoxicity and metabolism of these compounds. The parent compound 5NQ, its 8-methyl analogue (8Me-5NQ) and the 8-methylamino analogue (8NHMe-5NQ) each showed similar hypoxic selectivity (ratio of concentration × time for 90% kill for zero versus 20% oxygen of 13-18-, 30-69- and 1.2-1.4-fold respectively in the three cell lines tested (AA8 Chinese hamster ovary, EMT6/Ak mouse mammary tumour and FME human melanoma). The cytotoxicity and metabolism (covalent binding) of radiolabelled 8Me-5NQ was investigated in AA8 cultures over a range of oxygen tensions (0-95%). The oxygen tension in solution required for 50% inhibition of log cell kill or adduct formation observed under anoxia ($C_{50}$) was 0.01 and 0.02% oxygen respectively, suggesting that bioreductive alkylation is the mechanism of 8Me-5NQ toxicity. The $K$-value (oxygen concentration for cytotoxic potency equal to the mean of the potencies at zero and infinite oxygen) was similar (0.02% oxygen). Calculations based on measured rate constants for formation of the nitroradical anion of 8Me-5NQ and rates of radical loss through disproportionation or reaction with oxygen, predict a $K$-value for 8Me-5NQ of 0.025% oxygen, in good agreement with the experimentally determined value. Modelling of cell killing expected by the combination of 8Me-5NQ plus radiation suggested that tumour cells at intermediate oxygen tensions (0.01-1%) will be partially resistant to this treatment, and would limit the use of these 5-nitroquinolines in combination with radiation, unless sufficient drug could be delivered to cause extensive killing in the anoxic compartment.

It is widely recognised that there is a biologically significant subpopulation of cells at low oxygen concentrations in many human tumours (Thomlinson & Gray, 1955; Vaupel *et al.*, 1989; Chapman, 1991; Mueller-Klieser *et al.*, 1991; Vaupel *et al.*, 1991; Höckel *et al.*, 1993). The resistance of these predominantly non-cycling yet viable hypoxic cells to radiation (and probably to many chemotherapeutic agents) has led to the development of hypoxia-selective cytotoxins (HSCs), which are designed to be activated selectively in the absence of oxygen. Most HSCs are bioreductive drugs, their reductive activation in aerobic cells being inhibited by reoxidation of the initial one-electron reduction intermediate by oxygen (Wilson, 1992). Since they eliminate radioresistant hypoxic cells, HSCs have potential as tumour-selective radiation enhancers; this strategy has recently been termed 'bioreductive radiotherapy' (Brown, 1991).

The 4-alkylamino-5-nitroquinoline derivative 8Me-5NQ (Figure 1; R = Me) is a new and very selective HSC which was designed as a DNA-targeted bioreductive agent (Denny *et al.*, 1991). It is 60 times more potent against anoxic than aerobic cultures of Chinese hamster ovary AA8 cells (Denny *et al.*, 1992), this differential being similar to that for two HSCs currently under clinical investigation - RB 6145 and tirapazamine (SR 4233) - which have anoxic/aerobic differentials of about 20-fold and 70-fold respectively in the AA8 line (this laboratory, unpublished data). Despite this very high selectivity for anoxic cells *in vitro*, 8Me-5NQ has little anti-tumour activity in combination with radiation *in vivo* (Denny *et al.*, 1992), indicating that it is ineffective against radiobiologically hypoxic cells in tumours.

Evaluation of structure-activity relationships within the 4-alkylamino-5-nitroquinoline series has revealed a wide variation (1- to 60-fold) in hypoxia-selective cytotoxicity towards AA8 cells (Denny *et al.*, 1992). The basis of this variation in selectivity is not currently understood. This series therefore provides useful model compounds to investigate determinants of hypoxia-selective cytotoxicity within a congeneric series. As a first step in this process, three representative compounds with widely differing hypoxic selectivities against the AA8 cell line have been chosen for more detailed investigation. This study compares the highly selective 8Me-5NQ (selectivity 60-fold) with the parent compound (5NQ, Figure 1, R = H; selectivity 14-fold) and the 8-methylamino derivative (8NHMe-5NQ, Figure 1, R = NHMe), which has little selectivity for hypoxic AA8 cells (1.2-fold) (Denny *et al.*, 1992). The cytotoxicities of these compounds are compared in aerobic and anoxic cultures of three cell lines (AA8, the human melanoma line FME and the murine mammary carcinoma line EMT6/Ak) to determine whether the pattern of hypoxia-selective cytotoxicity observed in Chinese hamster AA8 cells is observed in cell lines derived from other species.

Most *in vitro* studies assess the hypoxia-selective cytotoxicity of bioreductive agents by comparing cytotoxicity under gas phases of 20% and 0% oxygen. While such comparisons are useful in screening new bioreductive drugs, they are of limited physiological significance since oxygen concentrations as high as 20% are not encountered in either normal tissues (typically 1-5% oxygen; Vanderkooi *et al.*, 1991) or tumours

**Figure 1** Structures of 4-alkylamino-5-nitroquinoline drugs investigated. 5NQ, R = H; 8Me-5NQ, R = Me; 8NHMe-5NQ, R = NHMe.

Correspondence: W.R. Wilson.
Received 7 February 1994; and in revised form 12 April 1994.

(Vaupel *et al.*, 1991). Conversely, there are probably few, if any, viable cells in tumours at 0% oxygen. Many tumour cells are at oxygen tensions which might be termed 'intermediate' (i.e. having a non-zero oxygen concentration, but hypoxic in comparison with normal tissues) and which must be taken into consideration in bioreductive radiotherapy. Studies with misonidazole (MISO) (Taylor & Rauth, 1982; Mulcahy, 1984), mitomycin C (Marshall & Rauth, 1986; Rauth & Marshall, 1990), porfiromycin (Marshall & Rauth, 1988), diaziquone (Rauth & Marshall, 1990; O'Brien *et al.*, 1990), RSU 1069 (Koch, 1993) and the cobalt complex SN 24771 (Wilson *et al.*, 1994) have indicated that for each of these drugs the concentration of oxygen required for 50% inhibition of the toxicity observed under anoxic conditions ($C_{50}$) is very low (*c.* 0.01% oxygen). In contrast, cells are not appreciably radiosensitised by oxygen until its concentration exceeds about 0.1% (Chapman *et al.*, 1974; Whillans & Hunt, 1982). It has been pointed out that tumour cells at intermediate oxygen tensions may therefore be too hypoxic to be radiosensitive, but too well oxygenated to activate bioreductive drugs efficiently, and may thus limit response to combinations of HSCs with radiation (Marshall & Rauth, 1988; Rauth & Marshall, 1990).

Clearly, investigation of new bioreductive drugs should consider the full range of physiological oxygen concentrations to assess the maximum differential which could be achieved *in vivo*, and to consider the contribution of cells at 'intermediate' oxygen to radioresistance in bioreductive was radiotherapy. This study reports the cytotoxicity and metabolic activation of 8Me-5NQ over a wide range of oxygen concentrations, and compares this with the less selective parent compound, 5NQ.

### Materials and methods

### *Drugs*

4-Alkylamino-5-nitroquinolines were prepared by published methods (Stefanska *et al.*, 1973; Denny *et al.*, 1992). Fresh drug solutions were prepared in culture medium [CM, alpha minimal essential medium (α-MEM) containing 5% (v/v) heat-inactivated foetal bovine serum (FBS) plus 100 IU $ml^{-1}$ penicillin and 100 μg $ml^{-1}$ streptomycin] for each experiment and were filter sterilised. Drug concentrations were checked by spectrophotometry, after dilution in 0.01 M hydrochloric acid, using extinction coefficients of 5,890 $M^{-1} cm^{-1}$ at 350 nm for 5NQ; 7110 $M^{-1} cm^{-1}$ at 352 nm for 8Me-5NQ; and 15,400 $M^{-1} cm^{-1}$ at 438 nm for 8NHMe-5NQ. 8Me-5NQ was labelled by catalytic exchange against $^3H_2O$ (Amersham International Ltd, Amersham, England) to prepare [*quinolinyl*-G-$^3$H]-8Me-5NQ. After purification by flash chromatography on an alumina column this had a radiochemical purity of 99.5% as determined by high-performance liquid chromatography (HPLC) and a specific activity of 34 GBq $mmol^{-1}$. A stock solution was prepared in 50% (v/v) ethanol and stored at −80°C.

### *Cell culture*

The AA8 cell line was obtained from L.H. Thompson (Lawrence Livermore Laboratory, Berkeley, CA, USA), the EMT6 line (designated EMT6/Ak in this laboratory) from I. Tannock (Ontario Cancer Institute, Toronto, Canada) and FME cells from K.M. Tveit (Norwegian Hydro Institute, Oslo, Norway). Cultures were maintained in logarithmic-phase growth in tissue culture flasks with weekly subculture by trypsinisation using CM (without antibiotics) as growth medium (the FBS concentration was 10% for FME). All cell lines were routinely shown to be free of mycoplasma contamination by fluorescence staining for cytoplasmic DNA (Chen, 1977). Bulk cultures of late log-phase AA8 cells (1.0–1.1 × $10^6$ cells $ml^{-1}$) were prepared in spinner flasks in CM containing 10% FBS by adjusting the cell density to 5 × $10^5$ $ml^{-1}$ 18 h before the experiment. Cells were harvested by centrifugation and resuspended in fresh CM to 5 × $10^6$ cells $ml^{-1}$. EMT6/Ak cells were prepared from multicellular spheroids grown in spinner flasks to a diameter of approximately 1 mm; these were dissociated by incubating for 30 min with Pronase (0.5 mg $ml^{-1}$) and DNAse I (1.0 mg $ml^{-1}$) at 37°C. Cells were centrifuged and resuspended in CM to provide a single-cell suspension at 2.5 × $10^6$ cells $ml^{-1}$. FME cells were prepared by growing monolayer cultures in 100-mm-diameter tissue culture dishes to plateau phase (4–5 × $10^5$ cells $ml^{-1}$), harvesting by trypsinisation (0.07% trypsin at 37°C for 10 min), pooling in CM containing DNAse I (0.2 mg $ml^{-1}$) and incubating at room temperature for 15 min before centrifugation and resuspension to 2.5 × $10^6$ cells $ml^{-1}$ in fresh CM.

### *Cytotoxicity*

Cytotoxicity was measured under controlled oxygenation in continuously gassed and stirred suspension cultures, using a modification of the method of Whillans and Rauth (1980). The apparatus comprised up to six gassing manifolds in a single 37°C waterbath over two nine-position magnetic stirrers, each manifold supplying up to nine glass Universal bottles (internal diameter 25 mm) containing cell suspensions which were stirred with glass-coated magnetic stir bars. Comparison of killing under aerobic (concentration of oxygen in gas phase ($C_g$) 20%) and anoxic ($C_g$ < 10 p.p.m. oxygen) conditions was made using 10 ml cultures at $10^6$ cells $ml^{-1}$ (AA8) or 5 × $10^5$ cells $ml^{-1}$ (EMT6/Ak and FME). These were prepared by equilibrating drug solutions (8 ml) in CM at 1.25 times the final required concentration at 37°C, using humidified gas mixtures containing 5% carbon dioxide (flowing through each vial at 100 ml $min^{-1}$), for 60 min prior to addition of cells (2 ml at five times the final cell density, equilibrated under the same gassing conditions). Samples were removed at intervals and colony formation assessed after incubation at 37°C for 8, 7 or 12 days (AA8, EMT6/Ak and FME respectively). Cytotoxicity to AA8 cells at a more extensive range of oxygen concentrations (typically with gas phases of 0, 0.2, 1, 5, 20 and 95% oxygen plus 5% carbon dioxide) was determined in the same manner using either $10^5$ or $10^6$ cells $ml^{-1}$, but the final culture volume was 6 ml and the lids were modified to allow insertion of an oxygen electrode.

### *Measurement of oxygen concentrations*

Oxygen was measured using a Clark-type oxygen electrode with a high-stability voltage supply and amplifier (Koch, 1991). In each experiment the electrode was calibrated in 20% oxygen, and the zero current measured from the anoxic drug exposure vial, which was determined in separate experiments to contain an oxygen concentration below the limit of detection of the electrode (*c.* 20 p.p.m. oxygen). The oxygen concentrations reported have been corrected for the current at zero oxygen (typically 10–20 pA). In experiments involving $C_g$ intermediate between 0 and 20%, the oxygen tension in solution ($C_s$) was monitored continuously in the 1% oxygen vial and was measured in all other vials at the end of the drug exposure. Values of $C_s$ are expressed as a percentage of that for CM saturated with 100% oxygen (1.01 mM).

### *Relationship between solution and gas-phase oxygen concentrations*

For continuously gassed, stirred cell suspensions such as the above, the relationship between the gas-phase oxygen concentration ($C_g$) and the steady-state concentration in solution at infinite time ($C_s$) is given by

$$C_s = C_g - R/k_1 \quad (1)$$

where $R$ is the rate of cellular oxygen consumption and $k_1$ is the geometry-dependent rate constant for the transport of oxygen across the solution–gas phase interface (Degn &

Wohlrab, 1971; Whillans & Rauth, 1980). $k_1$ was determined by measuring the rate of deoxygenation in the absence of cellular respiration by gassing CM (6 ml) with 95% nitrogen-5% carbon dioxide (<10 p.p.m. oxygen) under the above standard conditions. The value of $R$ for non-drug-treated AA8 cells was determined from the rate of loss of oxygen in aerobic AA8 cell suspensions ($5 \times 10^6$ cells $ml^{-1}$) in sealed respiration vials at 37°C.

*Macromolecular adduct formation by [$^3$H]8Me-5NQ*

Covalent binding of 8Me-5NQ metabolites to cellular macromolecules was determined under the same conditions as the AA8 cytotoxicity experiments except that [$^3$H]8Me-5NQ was added in a small volume (typically 20-30 µl) to 6 ml of pre-equilibrated cell suspension ($10^6$ $ml^{-1}$). At various times triplicate samples of $10^5$ cells were prepared from each vial by diluting 10-fold into ice-cold phosphate-buffered saline, and macromolecules were precipitated by adding cold 95% (v/v) ethanol (nine volumes). The samples were mixed by vortexing and stored at −20°C overnight. Precipitates were collected by filtration onto glass fibre filters (Whatman GF/C) and washed five times with 10 ml of cold 95% (v/v) ethanol and radioactivity determined by scintillation counting.

*Definition of $C_{50}$ and K values*

In experiments using a single drug concentration, the parameter used to quantify oxygen dependence was the $C_{50}$ value, defined as the $C_s$ required for 50% inhibition of the effect (yield of adducts or log cell kill) observed under anoxia. In experiments in which the drug concentration was adjusted to determine cytotoxic potency (as $1/CT_{10}$, where $CT_{10}$ is the concentration × time required to reduce survival to 10% of non-drug-treated controls), the oxygen dependence was quantified as the $K$-value, where $K$ is the $C_s$ when the potency is the arithmetic mean of that under anoxia and at infinite oxygen.

### Results

*Comparison of hypoxia-selective cytotoxicity in three cell lines*

The rate of killing of human melanoma FME cells by 8Me-5NQ was markedly enhanced under anoxic conditions relative to that under aerobic conditions ($C_g$ = 20%) (Figure 2). The hypoxia-selective cytotoxicity of 8Me-5NQ, the ratio of $CT_{10}$ values in aerobic and anoxic cultures, in the FME cell line was 66 ± 13 (Table I). Similar experiments were performed for 5NQ and 8NHMe-5NQ using FME cells (Table I). 5NQ, with a $CT_{10}$ ratio of 13 ± 2, had lower hypoxic selectivity than 8Me-5NQ, while the cytotoxicity of 8NHMe-5NQ towards FME cells was only marginally enhanced under hypoxia (selectivity ratio 1.4 ± 0.1).

To test whether the differences in hypoxic selectivity of 8Me-5NQ, 5NQ, and 8NHMe-5NQ were cell line dependent, similar experiments were performed using aerobic and anoxic stirred suspension cultures of AA8 and EMT6/Ak cells (Table I). In each of the cell lines 8NHMe-5NQ showed only marginal selectivity, and 8Me-5NQ was more selective than the parent 5NQ (Table I). Since the differences between these drugs were consistently observed in all three lines, further investigation of oxygen dependence was carried out using AA8 as a representative cell line.

*Bioreductive activation of 8Me-5NQ*

Labelling of the ethanol-insoluble (macromolecular) fraction of AA8 cells by [$^3$H]8Me-5NQ (40 µM) increased linearly with time under anoxic conditions (Figure 3), resulting in binding of 6.0% of the total radioactivity in anoxic cultures by 5 h. This labelling was almost completely inhibited under aerobic conditions, suggesting that 8Me-5NQ undergoes oxygen-inhibitable reductive activation to form a reactive species.

**Figure 2** Rate of killing of FME cells by 8Me-5NQ under aerobic (a; 20% oxygen) and anoxic (b) conditions.

*Intermediate oxygen tensions: characterisation of experimental system*

Drug cytotoxicity and metabolism were investigated over a wider range of oxygen concentrations by varying the oxygen content of the gas phase in the above stirred cell suspension system. The relationship between $C_g$ and $C_s$ was first investigated using non-drug-treated AA8 cell cultures (6 ml) at densities of $10^5$ and $10^6$ cells $ml^{-1}$ (Figure 4). The measured values of $C_s$ approached $C_g$ at high oxygen concentrations, but at lower values of $C_g$ the effects of cellular oxygen consumption in reducing $C_s$ were evident. At a density of $10^6$ cells $ml^{-1}$, this divergence became significant below a $C_g$ of about 5%, but at $10^5$ cells $ml^{-1}$ the effect of oxygen consumption was observed only below 1% oxygen.

The relationship between $C_g$ and $C_s$ at high oxygen concentrations was accounted for well by equation (1) using experimentally determined values of $k_1$[0.14 ± 0.01 (s.e.m.) $min^{-1}$] and $R$ [3.6 ± 0.1 × $10^{-17}$ (s.e.m.) mol $cell^{-1}$ $s^{-1}$], as shown by the dashed lines in Figure 4. However, at low oxygen concentrations the measured values of $C_s$ were higher than predicted by this equation, suggesting that $R$ decreases under these conditions.

**Table I** Aerobic and hypoxic cytotoxic potencies and hypoxic selectivity ratios for 5-nitroquinolines in stirred suspension cultures

| *Drug* | *Cell line* | *Aerobic potency $CT_{10}$ (μM h)*[a] | *Hypoxic potency $CT_{10}$ (μM h)*[a] | *Hypoxic selectivity ratio $CT_{10}$ (air/$N_2$)*[b] |
|---|---|---|---|---|
| 8Me-5NQ | FME | 1,855 ± 80 | 28 ± 3 | 66 ± 13 |
| | AA8 | 2,470 ± 600 | 30 ± 4 | 69 ± 10 |
| | EMT6/Ak | 1,215 ± 85 | 43 ± 1 | 30 ± 4 |
| 5NQ | FME | 350 ± 20 | 24 ± 1 | 13 ± 2 |
| | AA8 | 775 ± 40 | 52 ± 4 | 13 ± 1 |
| | EMT6/Ak | 510 ± 15 | 28 ± 2 | 18 ± 0.1 |
| 8NHMe-5NQ | FME | 2,245 ± 280 | 1,570 ± 230 | 1.4 ± 0.1 |
| | AA8 | 2,100 ± 600[c] | 2,180 ± 170 | 1.2 ± 0.1[c] |
| | EMT6/Ak | 2,645 ± 360 | 2,100 ± 265 | 1.2 ± 0.1 |

[a]Values are means ± s.e.m. for at least three determinations. [b]Values are means for intra-experiment comparisons in at least two independent experiments. The ratio in each experiment was determined from the pair of killing curves under air and nitrogen which gave the most similar rates of killing. [c]Data from Denny *et al.* (1992).

**Figure 3** Formation of ethanol-insoluble (macromolecular) adducts from [$^3$H]8Me-5NQ (40 μM) in AA8 cell cultures ($10^6$ cells $ml^{-1}$) under aerobic (○) and anoxic (●) conditions. Error bars are ranges for two independent experiments.

**Figure 4** Relationship between oxygen concentrations in the gas phase and in solution in stirred AA8 cell cultures at 0 (▽), $10^5$ (●) and $10^6$ (▲) cells $ml^{-1}$. Error bars are s.e.m. for three independent experiments.

*Oxygen-dependence of cytotoxicity and metabolic activation*

Killing of AA8 cells by 8Me-5NQ was examined over a range of oxygen concentrations, using a drug exposure sufficient to give measurable killing under anoxic conditions [surviving fraction (SF) = $1.2 \times 10^{-4}$ and $8.4 \times 10^{-3}$ in the two experiments; Figure 5]. Cell killing (determined as $-\log_{10}$ SF) was inhibited by very low concentrations of oxygen, with a $C_{50}$ value of *c.* 0.01% oxygen. A similar oxygen dependence experiment with 5NQ (50 μM, 2 h) provided a SF of $2.1 \times 10^{-3}$ under anoxia and also indicated a low $C_{50}$ value of *c.* 0.02% under these conditions (Figure 5, inset). Similar experiments with MISO (10 mM, 2 h, $10^6$ AA8 cells $ml^{-1}$) indicated an oxygen sensitivity very similar to that reported by Taylor and Rauth (1982), and gave a very low $C_{50}$ value of *c.* 0.005% oxygen (data not shown).

The oxygen dependence of covalent binding of 8Me-5NQ metabolites to macromolecules in AA8 cells was investigated under the same conditions (Figure 5). Formation of adducts at 2 h (anoxic values 3.7, 1.0 and 1.9 nmol $10^6$ cells$^{-1}$ in the three experiments) showed an oxygen dependence very similar to that for cell killing, with a $C_{50}$ value of *c.* 0.02% oxygen. There was some suggestion of residual covalent binding at high $C_s$ (>1%), although these values were only 2-fold greater than the 'non-specific' binding assessed from the ethanol-precipitable radioactivity in samples removed 1 min after addition of [$^3$H]8Me-5NQ.

In the above experiments, performed using a single drug concentration, killing under aerobic conditions was too low to measure. The rate of cell killing by 8Me-5NQ was determined in additional experiments using a range of drug concentrations (varying from 55 μM under anoxia to 1,950 μM in 95% oxygen) to enable quantitation of potency (as $1/CT_{10}$) over the full range of oxygen concentrations (Figure 6). Cytotoxic potency decreased progressively to a minimum value at 20% oxygen, with no further change when the oxygen concentration was increased to 95%. The *K*-value for 8Me-5NQ was *c.* 0.02% oxygen.

Analogous oxygen dependence experiments were performed with 5NQ by varying the drug concentration from 50 μM under anoxia to 1,000 μM in cultures equilibrated with 95% oxygen, to achieve a measurable rate of killing over the full range of $C_s$ values. Like 8Me-5NQ, no further inhibition of cytotoxicity was observed above 20% oxygen. The *K*-value for 5NQ (*c.* 0.1% oxygen) appeared to be greater than for 8Me-5NQ (*c.* 0.02% oxygen). However, the increase in $C_s$ with time after addition of drug to vials gassed at 1% oxygen was much more marked with 5NQ than with 8Me-5NQ under the conditions of these experiments (Figure 7) and would lead to an overestimation of the *K*-value. The increase in $C_s$ after addition of 5NQ was much less pronounced at a lower cell density of $10^5$ cells $ml^{-1}$ (Figure 7). At the lower density of $10^5$ cells $ml^{-1}$ extensive inhibition of killing by 5NQ (50 μM, 2 h) was observed at the lowest oxygen concen-

**Figure 5** Oxygen dependence of cell killing (filled symbols) and adduct formation (open symbols) after exposure of AA8 cells to drugs (55 μM 8Me-5NQ, 50 μM 5NQ) for 2 h. $C_s$ was determined at the end of the drug exposure period. Different symbols refer to separate experiments.

**Figure 6** Oxygen dependence of cytotoxic potency of 8Me-5NQ (●) and 5NQ (□). Error bars (s.d.) are shown for replicate determinations at $C_g = 0$ and 20% oxygen.

**Figure 7** Changes in oxygen concentration in solution (ordinate) during incubation of AA8 cells with bioreductive drugs ($C_g$ = 1% oxygen). ○, 8Me-5NQ, 55 μM, $10^6$ cells ml$^{-1}$; ▽, 5NQ, 50 μM, $10^6$ cells ml$^{-1}$. ▼, 5NQ, 50 μM, $10^5$ cells ml$^{-1}$.

tration tested ($C_g$ = 0.07%, $C_s$ = 0.016%, −log SF = 21% of anoxic value), indicating (by extrapolation) a $C_{50}$ value of c. 0.004% oxygen (data not shown).

### Discussion

*Lack of cell line dependence of hypoxia-selective cytotoxicity*

Some bioreductive drugs show marked variations in potency and hypoxia-selective cytotoxicity between cell lines owing to differences in repair or activity of bioactivating enzymes. For example, the potency and hypoxic selectivity of quinoidal HSCs such as mitomycin C and EO9 is dependent on the cell line, in part because of variations in expression of the two-electron reductase NAD(P)H:quinone oxidoreductase (DT diaphorase), which can activate such compounds in the presence of oxygen (Hoban et al., 1990; Dulhanty & Whitmore, 1991; Marshall et al., 1991a,b; Robertson et al., 1992).

The initial objective of this study was to assess whether the highly hypoxia-selective cytotoxicity of 8Me-5NQ in Chinese hamster AA8 cell cultures of 60-fold (Denny et al., 1991, 1992) is observed in other cell lines. The results demonstrate that this selectivity is not restricted to the AA8 line, with selectivity ratios of 66 and 30 observed in the human melanoma FME and murine carcinoma EMT6/Ak lines respectively (Table I). Investigation of two further 4-alkylamino-5-nitroquinolines, 5NQ and 8NHMe-5NQ, with lower hypoxic selectivity in the AA8 line (Denny et al., 1992) indicated the same pattern of selectivity (8Me-5NQ > 5NQ > 8NHMe-5NQ) in each of the three cell lines investigated. The magnitude of the hypoxic selectivity was similar in each line, although the air/nitrogen differential of 8Me-5NQ showed a minor variation (2-fold) between lines. Thus, based on this limited comparison, the determinants of the hypoxia-selective cytotoxicity of these 4-alkylamino-5-nitroquinolines appear to be largely independent of species and cell line. The AA8 cell line was therefore selected as representative for further investigation of the oxygen dependence of cytotoxicity and metabolic activation of these 5-nitroquinolines.

*Investigation of intermediate oxygen tensions*

The major technical difficulty in working with cell cultures at intermediate oxygen concentrations is that cellular respiration can appreciably lower $C_s$. The steady-state value of $C_s$ in this stirred system is a function of both the rate of transport of oxygen into the solution, $k_l$, and the rate of its consumption by cells, $R$, as described by equation (1) (Degn & Wohlrab, 1971; Whillans & Rauth, 1980). The relationships between $C_g$ and $C_s$ reported in Figure 4 agree closely with published data (Taylor & Rauth, 1982; Marshall et al., 1986), and are described well by equation (1) at high values of $C_g$. The discrepancy at low oxygen presumably reflects a decrease in $R$ as reported by others (Froese, 1962; Taylor & Rauth, 1982; Marshall et al., 1986).

*Inhibition of respiration by bioreductive drugs*

It is essential to monitor actual values of $C_s$ in experiments of this type since $R$ is not well defined at low oxygen concentrations, and can change with time in drug-treated cultures (Taylor & Rauth, 1982; O'Brien et al., 1990). Time-dependent increases in $C_s$ were observed with both of the 4-alkylamino-5-nitroquinolines investigated here (Figure 7), presumably as a result of progressive inhibition of respiration, as has been reported for MISO (Taylor & Rauth, 1982). When such changes are marked, as with 5NQ in AA8 cultures at $10^6$ cells ml$^{-1}$, it is difficult to define the oxygen dependence of killing with precision. The $C_s$ values reported here are for the end of the 2 h drug exposure period, and thus overestimate oxygen concentrations during treatment. The biasing effect of changing $C_s$ is illustrated by the reduction in the $C_{50}$ value for 5NQ cytotoxicity from 0.02 to *c.* 0.004% oxygen when the cell density is lowered from $10^6$ to $10^5$ cells ml$^{-1}$.

The observed inhibition of oxygen consumption by these bioreductive drugs has significance beyond the above methodological problem, since analogous changes in tumours could increase the diffusion distance of oxygen and hence cause significant reoxygenation. Drug-induced inhibition of respiration has been shown to radiosensitise multicellular spheroids through this mechanism (Biaglow & Durand, 1976, 1978; Durand, 1976). Although this process is potentially advantageous with radiosensitisers (since oxygen itself is a potent radiosensitiser), bioreductive activation of HSC would be compromised. The problem would be particularly severe for compounds with cytotoxicity restricted to regions at extremely low oxygen.

### *Covalent binding by metabolites of 8Me-5NQ*

Covalent binding to cellular macromolecules as a result of reductive metabolism has been reported for many nitro(hetero)aromatics (e.g. Chapman *et al.*, 1983; Wilson *et al.*, 1986; Liu *et al.*, 1992). The inhibition of 8Me-5NQ binding by oxygen strongly suggests that adduct formation depends on reductive activation. Further, the similarity of the oxygen dependence for cytotoxicity and binding to macromolecules ($C_{50}$ = 0.01 and 0.02% respectively) is evidence that this metabolic activation is responsible for the toxicity of 8Me-5NQ. This is consistent with studies with repair-deficient cell lines suggesting that cytotoxicity of this compound is due to formation of covalent DNA adducts (Denny *et al.*, 1991, 1992). The observation of some residual binding at high oxygen concentrations (Figure 5) has not been characterised adequately, but may indicate that a threshold level of adducts is required before cytotoxicity is observed and/or that an oxygen insensitive pathway contributes to activation.

### *Oxygen dependence of 8Me-5NQ and 5NQ cytotoxicity*

Both 5NQ and 8Me-5NQ have very low $C_{50}$ values for cytotoxicity, being in the order of 0.01% oxygen under standard conditions (2 h exposure at $10^6$ cells ml$^{-1}$, using a drug concentration giving *c.* 3–4 logs of killing under anoxia). These 4-alkylamino-5-nitroquinolines show a similar oxygen dependence to that reported for bioreductive drugs from a number of different drug classes: the nitro(hetero)arenes MISO (Taylor & Rauth, 1982; Mulcahy, 1984) and RSU 1069 (Koch, 1993); the quinones mitomycin C (Marshall & Rauth, 1986; Rauth & Marshall, 1990), porfiromycin (Marshall & Rauth, 1988) and diaziquone (O'Brien *et al.*, 1990; Rauth & Marshall, 1990); and the cobalt complex SN 24771 (Wilson *et al.*, 1994). In each of these cases 50% inhibition of the anoxic cell kill required *c.* 0.01% oxygen. As noted above, the true $C_{50}$ values for the 5-nitroquinolines in the present study may be even lower than the estimates at $10^6$ cells ml$^{-1}$, as is demonstrated by the very low $C_{50}$ for 5NQ (*c.* 0.004% oxygen) at a density of $10^5$ cells ml$^{-1}$. All of these compounds differ from the benzotriazine *N*-oxide tirapazamine (SR 4233), which requires substantially higher oxygen concentrations for inhibition of cytotoxicity (Koch, 1993).

Since the initial clinical use of bioreductive drugs is expected to be in combination with radiotherapy, it is appropriate to compare the oxygen dependence of these drugs with that for radiation. The relationship between radiosensitivity and oxygen concentration is described by a semiempirical hyperbolic function, the Alper and Howard-Flanders equation (Alper & Howard-Flanders, 1956), which contains two parameters: the OER (the differential in potency between infinite and zero oxygen) and the *K*-value. The latter is equal to the oxygen concentration giving a radiosensitivity (cytotoxic potency) that is the arithmetic mean of the values at zero and infinite oxygen. For an HSC a *K*-value can be defined in the same manner. The data of Figure 6 provide *K*-values of *c.* 0.02 and 0.1% oxygen for 8Me-5NQ and 5NQ respectively. The apparent difference between the two drugs may be an artifact due to the greater effect of 5NQ on cellular respiration.

8Me-5NQ resembles other nitro compounds in that its cytotoxicity is inhibited at very low oxygen concentrations. It is of interest to ask whether the observed *K*-value is consistent with the proposed mechanism of inhibition of activation by oxygen (Figure 8). At the *K*-value the concentration of $ArNO_2$ required for isoeffect ($[ArNO_2]_K$) is 2-fold higher than under anoxia ($[ArNO_2]_0$), but since the rate of killing is the same under both conditions the steady state concentration of $ArNO_2^{\bullet -}$ will be equal. Thus:

$$[ArNO_2]_0 = \tfrac{1}{2}[ArNO_2]_K \qquad (2)$$

$$[ArNO_2^{\bullet -}]_0 = [ArNO_2^{\bullet -}]_K \qquad (3)$$

where $[ArNO_2^{\bullet -}]_0$ is the concentration of $ArNO_2^{\bullet -}$ under anoxia and $[ArNO_2^{\bullet -}]_K$ is the concentration of $ArNO_2^{\bullet -}$ at the *K*-value. Assuming homogeneous kinetics (uniform distribution of $ArNO_2^{\bullet -}$ and oxygen) and steady-state conditions for $ArNO_2^{\bullet -}$, from Figure 8:

$$[ArNO_2^{\bullet -}]_K = \frac{-k_2[O_2] + (k_2^2[O_2]^2 + 8k_1k_3[ArNO_2]_K)^{\frac{1}{2}}}{4k_3} \qquad (4)$$

$$[ArNO_2^{\bullet -}]_0 = \frac{(4k_1k_3[ArNO_2]_K)^{\frac{1}{2}}}{4k_3} \qquad (5)$$

Solving equations (2–5) for $[O_2] = K$ gives:

$$K = \frac{(k_1k_3[ArNO_2]_K)^{\frac{1}{2}}}{k_2} \qquad (6)$$

The value of $k_1$ was determined from the rate loss of 8Me-5NQ in AA8 cultures under these conditions ($10^6$ cells ml$^{-1}$, 40 µM 8Me-5NQ, anoxia; Siim *et al.*, 1994). Assuming that there are two molecules of $ArNO_2^{\bullet -}$ formed for each molecule of $ArNO_2$ lost (Figure 8), this gave $k_1 = 1.4 \times 10^{-4}$ s$^{-1}$. Values of $8 \times 10^5$ M$^{-1}$ s$^{-1}$ and $2.5 \times 10^6$ M$^{-1}$ s$^{-1}$ for $k_2$ and $k_3$ respectively for 8Me-5NQ have been determined by pulse radiolysis (P. Wardman, personal communication). Using these values, plus $[ArNO_2]_K$ = 120 µM (from Figure 6), the predicted *K*-value for 8Me-5NQ is 256 nM (0.025% oxygen). The agreement between the measured (*c.* 0.02%) and predicted *K*-values for 8Me-5NQ supports the assumption that $ArNO_2^{\bullet -}$ and oxygen are isotropically distributed throughout the experimental system and suggests that the very low *K*-values for cytotoxicity of nitro compounds are accounted for by the rapid kinetics of back-oxidation of the radical by oxygen relative to further reduction via disproportionation.

In addition to *K*-values, plots of potency as a function of oxygen concentration provide information about the mechanism(s) of cytotoxicity. The potencies of 8Me-5NQ and 5NQ are not lowered further when the oxygen concentration is

**Figure 8** Reduction scheme for one-electron reduction of $ArNO_2$ to $ArNO_2^{\bullet -}$ and loss of $ArNO_2^{\bullet -}$ through disproportionation or reaction with oxygen. $k_1$ = rate constant for one-electron reduction of $ArNO_2$; $k_2$ = rate constant for reaction of $ArNO_2^{\bullet -}$ with oxygen; $k_3$ = rate constant for disproportionation of $ArNO_2^{\bullet -}$.

raised from 20 to 95% (Figure 6), so the selectivity ratios at supraphysiological oxygen are the same as the ratios for 0 and 20% oxygen (Table I). The failure to suppress cytotoxicity further at high oxygen concentrations is inconsistent with net reduction being the only mechanism of cytotoxicity. The high rates of reduction and one electron redox cycling of these 5-nitroquinolines (Siim et al., 1994) suggests that reactive oxygen species may contribute to cytotoxicity at high oxygen concentrations. This is consistent with the observed plateau in toxicity at high oxygen since when oxygen >> $K$ formation of the nitro radical will be rate limiting for superoxide formation, which will therefore not increase as oxygen is raised further. The higher aerobic potency (and lower hypoxic selectivity) of 5NQ than 8Me-5NQ (Table I) may reflect a greater contribution of oxygen species to cytotoxicity for 5NQ, which would be consistent with the higher rate of oxygen consumption induced in cyanide-inhibited AA8 cells by 5NQ than 8Me5NQ (Siim et al., 1994).

*Modelling of oxygen dependence of cell killing from drug + radiation*

Although convenient, parameters such as the $C_{50}$, $K$-value, or hypoxia-selective cytotoxicity ratio do not describe fully the oxygen dependence of bioreductive drugs. The interaction of bioreductive drugs with radiation in killing cells in tumours can only be assessed adequately by modelling this interaction using the estimated cell kill due to both agents alone over the full range of oxygen tensions. The results of this calculation are illustrated for the combination of 8Me-5NQ with a single dose of ionising radiation (2 Gy) in Figure 9, using the oxygen dependence of cell kill by 8Me-5NQ reported in Figure 5 and published oxygen dependence data for the radiosensitivity of CHO cells (Whillans & Hunt, 1982), and assuming an OER of 2.5 at a dose of 2 Gy. SF values due to radiation were calculated using the linear-quadratic model assuming $\alpha = 0.3\,Gy^{-1}$ and $\beta = 0.03\,Gy^{-2}$ at 20% oxygen, these parameters being typical for human cells (Fertil & Malaise, 1985). If it is assumed that anoxic cell killing due to the drug is the same as that achieved by radiation in the oxic subpopulation (SF = 0.5), then cells at intermediate oxygen are spared by this combination. However, if sufficient drug can be delivered to kill four logs of anoxic cells, then the resistance at intermediate oxygen disappears (Figure 9). Such extensive killing is unlikely if hypoxia is predominantly the result of fluctuating tumour blood flow; cells which are radioresistant because a vessel has recently closed may reoxygenate because of reopening of the vessel, thus terminating hypoxic drug exposure. However, it is clear that the magnitude of the problem at intermediate oxygen concentrations depends critically on the *absolute* cell kill which can be achieved with a bioreductive drug, and cannot be assessed adequately by comparing the *relative* sensitivities of drug and radiation to oxygen. The importance of using absolute rather than relative parameters to describe modification of radiation response has been emphasised recently (Koch & Skov, 1992).

*Implications for therapeutic use*

This study has identified two problems which may limit the activity of 4-alkylamino-5-nitroquinoline drugs in bioreductive radiotherapy. Inhibition of respiration by these compounds could increase diffusion of oxygen and eliminate the microenvironments best able to activate the compound. Further, cells at 'intermediate' oxygen tensions will be relatively resistant to both radiation and the drug. The extent of this problem will depend on the drug concentration which can be achieved in tumours. The maximum tolerated dose of 8Me-5NQ in $C_3H$/HeN mice is only 100 μmol kg$^{-1}$ (Denny et al., 1992), suggesting that concentrations in tumours of >100 μM are not likely to be achieved. Since a concentration × time of c. 50 μM h is required for appreciable killing of even fully anoxic cells by 8Me-5NQ *in vitro* (Figure 6), extensive killing of these cells in mouse tumours is unlikely. These problems, along with restricted tissue penetration imposed by non-covalent DNA binding (Durand, 1986; Wilson & Denny, 1992), may account for the disappointing lack of activity of 5NQ and 8Me-5NQ as tumour radiosensitisers *in vivo* (Denny et al., 1992; Wilson et al., 1992).

The investigation of these model compounds has pointed to the difficulty of *de novo* design of bioreductive drugs. Development of compounds with high differential toxicity in standard *in vitro* screens is a first step, but there are many potential problems which may preclude *in vivo* activity. There is thus an obvious need to evaluate new candidate compounds *in vivo* at an early stage in any bioreductive drug development programme.

**Figure 9** Combination of 8Me-5NQ with radiation at a range of oxygen concentrations. ●; low-dose 8Me-5NQ (anoxic SF = 0.5); ■, high-dose 8Me-5NQ (anoxic SF = $10^{-4}$); ○, radiation only (2 Gy); solid line without symbols, low-dose 8Me-5NQ plus radiation; dashed line, high-dose 8Me-5NQ plus radiation.

This study was supported by the Health Research Council of New Zealand, the Cancer Society of New Zealand and the National Cancer Institute (contract CM 07321). B.G.S. was the recipient of a Postgraduate Scholarship from the University Grants Committee of New Zealand. We thank S. Cliffe for assistance with the oxygen electrode measurements and C.J. Koch and A.M. Rauth for helpful discussions on the oxygen concentration dependence studies.

**Abbreviations:** $C_g$, concentration of oxygen in the gas phase; $C_s$, concentration of oxygen in solution; CM, culture medium [α-MEM containing 5% (v/v) heat-inactivated FBS plus 100 IU ml$^{-1}$ penicillin and 100 μg ml$^{-1}$ streptomycin]; $CT_{10}$, concentration × time to reduce survival to 10%; FBS, foetal bovine serum; HSC, hypoxia-selective cytotoxin; MISO, misonidazole; OER, oxygen enhancement ratio; s.d., standard deviation; s.e.m., standard error of the mean; SF, surviving fraction.

### References

ALPER, T. & HOWARD-FLANDERS, P. (1956). The role of oxygen in modifying the radiosensitivity of *E. coli* B. *Nature*, **178**, 978–979.

BIAGLOW, J.E. & DURAND, R.E. (1976). The effects of nitrobenzene derivatives on oxygen utilization and radiation response of an *in vitro* tumor model. *Radiat. Res.*, **65**, 529–539.

BIAGLOW, J.E. & DURAND, R.E. (1978). The enhanced radiation response of an *in vitro* tumour model by cyanide released from hydrolysed amygdalin. *Int. J. Radiat. Biol.*, **33**, 397-401.

BROWN, J.M. (1991). Targeting bioreductive drugs to tumours: is it necessary to manipulate blood flow? *Int. J. Radiat. Biol.*, **60**, 231-236.

CHAPMAN, J.D., DUGLE, D.L., REUVERS, A.P., MEEKER, B.E. & BORSA, J. (1974). Studies on the radiosensitizing effect of oxygen in Chinese hamster cells. *Int. J. Radiat. Biol.*, **26**, 383-389.

CHAPMAN, J.D., BAER, K. & LEE, J. (1983). Characteristics of the metabolism-induced binding of misonidazole to hypoxic mammalian cells. *Cancer Res.*, **43**, 1523-1528.

CHAPMAN, J.D. (1991). Measurement of tumor hypoxia by invasive and non-invasive procedures: a review of recent clinical studies. *Radiother. Oncol.*, **20** (Suppl.), 13-19.

CHEN, T.R. (1977). *In situ* detection of mycoplasma contamination in cell cultures by fluorescent Hoechst 33258 stain. *Exp. Cell Res.*, **104**, 255-262.

DEGN, H. & WOHLRAB, H. (1971). Measurement of steady-state values of respiration rate and oxidation levels of respiratory pigments at low oxygen tensions. A new technique. *Biochim. Biophys. Acta*, **245**, 347-355.

DENNY, W.A., WILSON, W.R., ATWELL, G.J., BOYD, M., PULLEN, S.M. & ANDERSON, R.F. (1991). Nitroacridines and nitroquinolines as DNA-affinic hypoxia-selective cytotoxins. In *Selective Activation of Drugs by Redox Processes*, Adams, G.E., Breccia, A., Fielden, E.M. & Wardman, P. (eds) pp. 149-158. Plenum Press: New York.

DENNY, W.A., ATWELL, G.J., ROBERTS, P.B., ANDERSON, R.F., BOYD, M., LOCK, C.J.L. & WILSON, W.R. (1992). Hypoxia-selective agents. 6. 4-(Alkylamino)nitroquinolines: a new class of hypoxia-selective cytotoxins. *J. Med. Chem.*, **35**, 4832-4841.

DULHANTY, A.M. & WHITMORE, G.F. (1991). Chinese hamster ovary cell lines resistant to mitomycin C under aerobic but not hypoxic conditions are deficient in DT-diaphorase. *Cancer Res.*, **51**, 1860-1865.

DURAND, R.E. (1976). Adriamycin: a possible indirect radiosensitizer of hypoxic tumor cells. *Radiology*, **119**, 217-222.

DURAND, R.E. (1986). Chemosensitivity of V79 spheroids: drug delivery and cellular microenvironment. *J. Natl Cancer Inst.*, **77**, 247-252.

FERTIL, B. & MALAISE, E.P. (1985). Intrinsic radiosensitivity of human cell line is correlated with radioresponsiveness of human tumors: analysis of 101 published survival curves. *Int. J. Radiat. Oncol. Biol. Phys.*, **11**, 1699-1707.

FROESE, G. (1962). The respiration of ascites tumour cells at low oxygen concentrations. *Biochim. Biophys. Acta*, **57**, 509-519.

HOBAN, P.R., WALTON, M.I., ROBSON, C.N., GODDEN, J., STRATFORD, I.J., WORKMAN, P., HARRIS, A.L. & HICKSON, I.D. (1990). Decreased NADPH:cytochrome P-450 reductase activity and impaired drug activation in a mammalian cell line resistant to mitomycin C under aerobic but not hypoxic conditions. *Cancer Res.*, **50**, 4692-4697.

HÖCKEL, M., KNOOP, C., SCHLENGER, K., VORNDRAN, B., BAUßMANN, E., MITZE, M., KNAPSTEIN, P.G. & VAUPEL, P. (1993). Intratumoral $pO_2$ predicts survival in advanced cancer of the uterine cervix. *Radiother. Oncol.*, **26**, 45-50.

KOCH, C.J. (1991). Polarographic oxygen sensor. US Patent 5030036. July, 1991.

KOCH, C.J. & SKOV, K.A. (1992). Comparisons of cellular radiation response using absolute rather relative parameters. *Radiat. Res.*, **132**, 40-49.

KOCH, C.J. (1993). Unusual oxygen concentration dependence of toxicity of SR-4233, a hypoxic cell toxin. *Cancer Res.*, **53**, 3992-3997.

LIU, Y.-Y., LU, A.Y.H., STREANS, R.A. & CHIU, S.-H.L. (1992). *In vivo* covalent binding of [$^{14}$C]trinitrotoluene to proteins in the rat. *Chem. Biol. Interact.*, **82**, 1-19.

MARSHALL, R.S. & RAUTH, A.M. (1986). Modification of the cytotoxic activity of mitomycin C by oxygen and ascorbic acid in Chinese hamster ovary cells and a repair-deficient mutant. *Cancer Res.*, **46**, 2709-2713.

MARSHALL, R.S., KOCH, C.J. & RAUTH, A.M. (1986). Measurement of low levels of oxygen and their effect on respiration in cell suspensions maintained in an open system. *Radiat. Res.*, **109**, 91-101.

MARSHALL, R.S. & RAUTH, A.M. (1988). Oxygen and exposure kinetics as factors influencing the cytotoxicity of porfiromycin, a mitomycin C analogue, in Chinese hamster ovary cells. *Cancer Res.*, **48**, 5655-5659.

MARSHALL, R.S., PATERSON, M.C. & RAUTH, A.M. (1991*a*). Studies on the mechanism of resistance of Mitomycin C and Porfiromycin in a human cell strain derived from a cancer-prone individual. *Biochem. Pharmacol.*, **41**, 1351-1360.

MARSHALL, R.S., PATERSON, M.C. & RAUTH, A.M. (1991*b*). DT-diaphorase activation and mitomycin C sensitivity in non-transformed cell strains from members of a cancer-prone family. *Carcinogenesis*, **12**, 1175-1180.

MUELLER-KLIESER, W., SCHLENGER, K.-H., WALENTA, S., GROSS, M., KARBACH, U., HOECKEL, M. & VAUPEL, P. (1991). Pathophysiological approaches to identifying tumor hypoxia in patients. *Radiother. Oncol.*, **20** (Suppl.), 21-28.

MULCAHY, R.T. (1984). Effect of oxygen on misonidazole chemosensitization and cytotoxicity *in vitro*. *Cancer Res.*, **44**, 4409-4413.

O'BRIEN, P.J., KAUL, H.K. & RAUTH, A.M. (1990). Differential cytotoxicity of diaziquone toward Chinese hamster ovary cells under hypoxic and aerobic exposure conditions. *Cancer Res.*, **50**, 1516-1520.

RAUTH, A.M. & MARSHALL, R.S. (1990). Mechanisms of activation of mitomycin C and AZQ in aerobic and hypoxic mammalian cells. In *Selective Activation of Drugs by Redox Processes*, Adams, G.E. (ed.) pp. 113-123. Plenum Press: New York.

ROBERTSON, N., STRATFORD, I.J., HOULBROOK, S., CARMICHAEL, J. & ADAMS, G.E. (1992). The sensitivity of human tumor cells to quinone bioreductive drugs - what role for DT-diaphorase? *Biochem. Pharmacol.*, **44**, 409-412.

SIIM, B.G., ATWELL, G.J. & WILSON, W.R. (1994). Metabolic and radiolytic reduction of 4-alkylamino-5-nitroquinoline bioreductive drugs: relationship to selectivity for hypoxic cells. *Biochem. Pharmacol.* (in press).

STEFANSKA, B., ZIRRA, J.A., PERYT, J., KAMINSKI, K. & LEDOCHOWSKI, A. (1973). Research on tumour inhibiting compounds. LII. 5- and 8-nitro-4-aminoquinoline derivatives. *Rocz. Chem.*, **47**, 2339-2343.

TAYLOR, Y.C. & RAUTH, A.M. (1982). Oxygen tension, cellular respiration, and redox state as variables influencing the cytotoxicity of the radiosensitizer misonidazole. *Radiat. Res.*, **91**, 104-123.

THOMLINSON, R.H. & GRAY, L.H. (1955). The histological structure of some human lung cancers and possible implications for radiotherapy. *Br. J. Cancer*, **9**, 539-549.

VANDERKOOI, J.M., ERECINSKI, M. & SILVER, I.A. (1991). Oxygen in mammalian tissue: methods of measurement and affinities of various reactions. *Am. J. Physiol.*, C1131-C1150.

VAUPEL, P., KALLINOWSKI, F. & OKUNIEFF, P. (1989). Blood flow, oxygen and nutrient supply and metabolic microenvironment of human tumors: a review. *Cancer Res.*, **49**, 6449-6465.

VAUPEL, P., SCHLENGER, K., KNOOP, C. & HÖCKEL, M. (1991). Oxgenation of human tumours: evaluation of tissue oxygen distribution in breast cancers by computerized $O_2$ tension measurements. *Cancer Res.*, **51**, 3316-3322.

WHILLANS, D.W. & RAUTH, A.M. (1980). An experimental and analytical study of oxygen depletion in stirred cell suspensions. *Radiat. Res.*, **84**, 97-114.

WHILLANS, D.W. & HUNT, J.W. (1982). A rapid-mixing comparison of the mechanisms of radiosensitization by oxygen and misonidazole. *Radiat. Res.*, **90**, 126-141.

WILSON, W.R., DENNY, W.A., STEWART, G.M., FENN, A. & PROBERT, J.C. (1986). Reductive metabolism and hypoxia-selective toxicity of nitracrine. *Int. J. Radiat. Oncol. Biol. Phys.*, **12**, 1235-1238.

WILSON, W.R. (1992). Tumour hypoxia: challenges for cancer chemotherapy. In *Cancer Biology and Medicine*, Vol. 3, Waring, M.J. & Ponder, B.A.J. (eds) pp. 87-131. Kluwer Academic Publishers: Lancaster.

WILSON, W.R. & DENNY, W.A. (1992). DNA-binding nitroheterocycles as hypoxia-selective cytotoxins. In *Radiation Research, a Twentieth-century Perspective*, Vol. 2, Dewey, W.C., Edington, M., Fry, R.J.M., Hall, E.J. & Whitmore, G.F. (eds) pp. 796-801. Academic Press: San Diego.

WILSON, W.R., SIIM, B.G., DENNY, W.A., VAN ZIJL, P.L., TAYLOR, M.L., CHAMBERS, D.M. & ROBERTS, P.B. (1992). 5-Nitro-4-(*N,N*-dimethylaminopropylamino)quinoline (5-Nitraquine), a new DNA-affinic hypoxic cell radiosensitizer and bioreductive agent: comparison with Nitracrine. *Radiat. Res.*, **131**, 257-265.

WILSON, W.R., MOSELEN, J.W., CLIFFE, S., DENNY, W.A. & WARE, D.C. (1994). Exploiting tumor hypoxia through bioreductive release of diffusible cytotoxins: the cobalt(III)-nitrogen mustard complex SN 24771. *Int. J. Radiat. Oncol. Biol. Phys.*, **29**, 323-327.